# Peptide CCAT1-70aa promotes hepatocellular carcinoma proliferation and invasion via the MAPK/ERK pathway

**DOI:** 10.1515/med-2025-1206

**Published:** 2025-08-19

**Authors:** Xianjian Wu, Ruifeng Liang, Guoman Liu, Quan Fang, Zuoming Xu, Wenchuan Li, Chuan Tan, Jian Pu

**Affiliations:** Department of Hepatobiliary Surgery, The Affiliated Hospital of Youjiang Medical University for Nationalities, Baise, Guangxi, 533000, China; Graduate College, Youjiang Medical University for Nationalities, Baise, Guangxi, 533000, China; Guangxi Clinical Medical Research Center of Hepatobiliary Disease, Baise, People’s Republic of China; Department of Hepatobiliary Surgery, The Affiliated Hospital of Youjiang Medical University for Nationalities, Zhongshan 2nd Road, Baise, Guangxi, 533000, China

**Keywords:** liver cancer, encoding peptides, MAPK signaling, tumor growth, metastatic potential

## Abstract

**Objective:**

Peptide-encoding roles of lncRNAs are emerging in cancer biology. This study explores the function of the CCAT1-70aa peptide in hepatocellular carcinoma (HCC) and its underlying mechanisms.

**Methods:**

Immunohistochemistry was used to detect CCAT1-70aa expression in HCC and adjacent tissues. An expression vector verified CCAT1’s role in encoding CCAT1-70aa. Cell counting kit-8 and Transwell assays assessed the effects of CCAT1-70aa on HCC cell proliferation and invasion. Small-interfering RNAs (siRNAs) targeting CCAT1 were transfected into HCC cells to examine CCAT1-70aa expression. The role of the MAPK/ERK pathway was confirmed via Western blot and the ERK inhibitor FR180204.

**Results:**

CCAT1-70aa was significantly upregulated in HCC tissues, correlating with tumor stage, serum alpha-fetoprotein levels, and vascular invasion. siRNA-mediated CCAT1 silencing reduced CCAT1-70aa expression, supporting that CCAT1-70aa is translated from lncRNA CCAT1. CCAT1-70aa, a 70-amino acid peptide, enhanced proliferation and invasion, activating the MAPK/ERK pathway, with its effects mitigated by ERK inhibition.

**Conclusion:**

The CCAT1-70aa peptide is overexpressed in HCC and linked to aggressive tumor characteristics. It promotes proliferation and invasion via the MAPK/ERK pathway, providing insights for HCC diagnosis and treatment strategies.

## 1 Introduction

Hepatocellular carcinoma (HCC), also referred to as liver cancer, is the second most common cancer in China and the third highest cause of cancer-related death globally with a mortality rate of 8.2% [1]. Despite the relatively favorable prognosis with early treatment and a 5-year survival rate exceeding 72%, the annual incidence of HCC in China remains high, reaching 350,000 and accounting for 50% of newly diagnosed cases worldwide [2]. Unfortunately, many HCC patients are diagnosed at advanced stages due to factors, such as tumor size, spread, and co-existing liver disease. This situation often results in suboptimal outcomes [2]. The lack of specific symptoms in the early stages, along with the absence of effective screening mechanisms and early diagnostic methods, leads to many patients presenting with late-stage HCC at their initial clinical consultation [3]. Consequently, there is an urgent need for new diagnostic and therapeutic strategies to improve the prognosis for HCC. In terms of etiology, HCC usually evolves from chronic liver disease, primarily associated with hepatitis B or C virus infection, alcohol consumption, or metabolic syndrome [4]. Although HCC poses a severe threat to human health, the cellular and molecular mechanisms underlying the initiation and progression of HCC are still poorly understood [5]. A more in-depth elucidation of the key molecules and their regulatory mechanisms involved in HCC is crucial in developing more effective treatment strategies for this deadly disease [6].

Long non-coding RNAs (lncRNAs) are RNA molecules longer than 200 nucleotides that lack protein-coding capacity. They have been reported to play significant regulatory roles in various types of cancers [7]. They often participate in disease regulation through mechanisms such as direct binding to specific targets [8], serving as sponges for microRNAs (miRNAs) [9], or through epigenetic processes [10]. The lncRNA colon cancer-associated transcript 1 (lncRNA CCAT1) has been proven to promote various types of cancers, including colorectal cancer, gastric cancer, and pancreatic cancer [11,12,13]. The role of lncRNA CCAT1 in HCC has received extensive investigation in recent literature, for example, recent research indicates that lncRNA CCAT1 is upregulated in HCC and promotes the development of HCC through interactions with let-7 [14], miR-30c-2-3p [15], and miR-222-5p [16]. These pathways include enhancing the resistance of HCC to chemotherapy [17], promoting the proliferation and invasion of HCC cells [18], and promoting autophagy in HCC by regulating ATG7 [19]. Moreover, the aberrant expression of CCAT1 is regulated by c-Myc and can predict the prognosis of HCC [20]. Additionally, CCAT1 regulates the expression of the IRF5 gene by adsorbing miR-375-3p [21], regulates the expression of cell cycle-dependent kinase 1 as a competitive endogenous RNA of miR-490-3p [22], and is activated by tumor-associated macrophages via the CCAT1/let-7b/HMGA2 pathway, thus promoting HCC [23].

Previous studies from our research group have shown that the lncRNA CCAT1 promotes the progression of HCC by enhancing EGFR signaling, as well as through the miR-222-5p/CYLD pathway [16]. Recent studies have indicated that certain lncRNAs possess an open reading frame (ORF) and can encode peptides to participate in disease regulation [24,25]. Our experimental data demonstrate that lncRNA CCAT1 encodes a peptide, confirmed as CCAT1-70aa through *in vitro* translation assays. The function and mechanism of the peptide CCAT1-70aa in HCC remain unexplored and warrant further investigation. In this study, we aim to elucidate how lncRNA CCAT1 encodes the peptide CCAT1-70aa and how this peptide can promote the proliferation and metastasis of HCC through the mitogen-activated protein kinase (MAPK)/extracellular signal-regulated kinase (ERK) pathway. This study aims to deepen our understanding of the molecular mechanisms driving HCC development and progression.

## 2 Materials and methods

### 2.1 Clinical samples

From January 2022 to December 2022, 88 cases of HCC and adjacent tissues were collected from the Affiliated Hospital of the Right River Institute of Ethnic Medicine. The inclusion criteria for subjects included (1) absence of preoperative radiotherapy, chemotherapy, or other clinical adjuvant therapies, such as radiofrequency ablation, and (2) HCC diagnosis was confirmed by two or more pathologists. Exclusion criteria included (1) subjects with serious medical conditions, such as heart disease, kidney disease, or other malignant tumors; (2) subjects who had previously undergone radiation therapy or chemotherapy; (3) conditions that might affect their participation in the study, including mental illness, drug or alcohol abuse, etc.; and (4) inability or unwillingness to sign the informed consent. Tumor pathological staging followed the TNM classification of the International Union Against Cancer. Tumor differentiation was assessed using WHO grading criteria. Other information was collected according to routine clinical records.

### 2.2 Cell culture

The HCC cell lines, Huh-7 and Hep3B, were purchased from Wuhan Procell Life Technology Co., Ltd. The cells were cultured in Dulbecco’s Modified Eagle’s Medium supplemented with 10% fetal bovine serum and 1% penicillin and streptomycin. The cells were cultured in an incubator (3111, Thermo Fisher) set at 37°C with 95% air and 5% CO_2_. The medium was changed 2–3 times per week with a subculture ratio of 1:3. When treating with FR180204 (Sigma-Aldrich, F3672), a concentration of 20 nM was used for 24 h.

### 2.3 Anti-CCAT1-70aa antibody preparation

The CCAT1-70aa antibody was prepared by SinoBiologica Inc. (Beijing, China). Briefly, a KLH-coupled peptide corresponding to the CCAT1-70aa sequence was synthesized based on the following primers: forward primer 5′-ATGGTTGAGAAAAGTCATC-3′ and reverse primer 5′-AAGTTTTCCTGTGTGGCTC-3′. Polyclonal antibodies against the CCAT1-70aa peptide were obtained from inoculated rabbits.

### 2.4 Immunohistochemistry

Tissues were fixed with 4% paraformaldehyde, embedded in paraffin, and sectioned to a thickness of 4 µm. After deparaffinization, antigen retrieval was performed by high-pressure boiling. Immunohistochemical staining was conducted using a CCAT1-70aa-specific antibody. Visualization was achieved using 3,3′-diaminobenzidine and the intensity of positive staining was quantified to conduct a statistical analysis. The stained sections were examined under a light microscope at magnifications of 200× to assess the distribution and intensity of positive staining.

### 2.5 Vector construction and cell transfection

The peptide sequence encoded by the lncRNA CCAT1 was predicted using the CPC 2.0 database (http://cpc2.gao-lab.org/). The CCAT1-70aa construct was designed with a 3 × Flag tag at the N-terminus. The full-length sequence of CCAT1-70aa was synthesized according to the gene sequence and cloned into the GV358 vector (element sequence: Ubi-MCS-3FLAG-SV40-EGFP-IRES-puromycin). The vector construction followed the methods outlined in reference [26]. The choice of the GV358 vector was made based on several factors: its ubiquitin promoter enables high expression efficiency in mammalian cells, facilitating robust and stable expression of CCAT1-70aa in HCC cells; the inclusion of EGFP allows for visual tracking of transduction efficiency; and the puromycin resistance marker enables antibiotic selection of successfully transduced cells. Additionally, this vector is compatible with the generation of mutant constructs, essential for studying both wild-type and mutant forms of CCAT1-70aa. After sequencing verification, the vector was co-transfected with helper plasmids into 293T cells. The supernatant was collected, concentrated, and lentivirus was obtained. A sequence from the 5′UTR of lncRNA CCAT1 was added to generate a 5′UTR-70aa sequence. By mutating the start codon ATG to ATT in the predicted ORF sequence, a 5′UTR-70aa-MUT sequence was obtained. A corresponding lentiviral vector was constructed in the same way as the CCAT1-70aa overexpression lentivirus. Hep3B and Huh-7 cells were transduced with the lentivirus in the presence of polybrene at a multiplicity of infection of 10. Transduction efficiency was evaluated under a fluorescence microscope after 96 h and followed by further experiments. An empty GV358 vector containing only the 3× Flag tag, without the CCAT1-70aa insert, was used as the negative control (NC).

For gene knockdown experiments, small-interfering RNAs (siRNAs) targeting CCAT1 were synthesized and transfected into Huh-7 cells using Lipofectamine 3000 (Invitrogen, USA) according to the manufacturer’s protocol. The sequences of the siRNAs were as follows: si-CCAT1#1 (AGCCTTGTAGAAACACTATCA), si-CCAT1#2 (ATCTGATTTGACTAAACATGA), and a non-targeting siRNA control (si-NC, UUCUCCGAACGAGUCACGUTT). The siRNAs and si-NC were synthesized by Guangzhou Anernor Biotechnology Co., Ltd. Huh-7 cells were harvested 48 h post-transfection for subsequent analysis.

### 2.6 Reverse transcription polymerase chain reaction (RT-PCR)

Total RNA was extracted from Huh-7 cells using TRIzol reagent (Invitrogen, USA) and reverse transcribed into cDNA using the PrimeScript™ RT reagent kit (Takara, Japan). RT-PCR was performed using 2× Taq PCR Master Mix (Takara, Japan) with specific primers for CCAT1-70aa (forward primer: 5′-CATTGGGAAAGGTGCCGAGA-3′, reverse primer: 5′-ACGCTTAGCCATACAGAGCC-3′) and GAPDH (forward primer: 5′-CCAGGTGGTCTCCTCTGA-3′, reverse primer: 5′-GCTGTAGCCAAATCGTTGT-3′). Band intensities were quantified using ImageJ software (NIH, USA), and relative expression levels of CCAT1-70aa were calculated using the 2^−ΔΔCt^ method, with GAPDH as an internal control.

### 2.7 Western blot

Cells were washed three times with phosphate-buffered saline (PBS) and lysed in cell lysis buffer, followed by incubation at 4°C for 20 min. The lysate was centrifuged at 14,000 rpm for 10 min, and the supernatant was collected and stored at −80°C. Protein concentration was determined using a BCA assay (P0012, Beyotime). Samples were diluted 15-fold and then mixed with 200 μl of BCA working solution, followed by incubation at 37°C for 30 min. Absorbance was measured at 562 nm, and protein concentration in the samples was calculated using a standard curve. The acrylamide gel plates separating gel and stacking gel were prepared. 20 μg of the sample was loaded and electrophoresis was performed at 100 V for approximately 1.5 h. After electrophoresis, the gel was transferred onto a PVDF membrane at 300 mA for 39 min. After blocking the membrane, primary antibodies against CCAT1-70aa, Flag (Abcam, ab205606, 1:2,000), ERK (Abcam, ab32537, 1:1,000), p-ERK (Abcam, ab194770, 1:1,000), Ras (Abcam, ab52939, 1:5,000), c-Raf (Abcam, ab236003, 1:2,000), p-c-Raf (Abcam, ab150365, 1:2,000), MEK (Abcam, ab32576, 1:10,000), p-MEK (Abcam, ab96379, 1:2,000), and GAPDH (Abcam, ab181602, 1:10,000) were added and incubated overnight at 4°C. After washing, the secondary antibody (Abcam, ab6721, 1:10,000) was added and incubated at room temperature for 40 min. Equal volumes of detection reagents A and B were mixed and added to the PVDF membrane (Millipore) for film development. When the bands became clear, the membrane was exposed and fixed for 10 min using the developing solution. The intensity of the bands was analyzed using ImageJ software (V1.8.0, National Institutes of Health) after scanning the film. GAPDH was used as an internal reference to calculate relative protein expression.

### 2.8 Immunofluorescence

Cell smear slides were prepared and incubated at 60°C for 30 min. The slides were deparaffinized in xylene and rehydrated in a descending ethanol series, followed by a distilled water rinse. Antigen retrieval was conducted using citrate buffer and microwave heating, before washing with PBS. Endogenous peroxidase activity was blocked with 3% H_2_O_2_, and slides were washed with PBS. An optional step involved treating the slides with an autofluorescence quencher A prior to immunolabeling. Serum blocking was performed, followed by incubation with the primary antibody, recombinant Anti-DDDDK tag (Binds to FLAG^®^ tag sequence) antibody (Abcam, ab205606, 1:100) overnight at 4°C. Slides were then washed and incubated with the fluorescent secondary antibody (A0516, Beyotime, 1:200) at 37°C, followed by further washing. Operations from this point were performed in the dark. 4′,6-Diamidino-2-phenylindole (DAPI) was used for nucleus staining with subsequent PBS washes. The slides were treated with autofluorescence quencher B. Finally, the slides were dried slightly, sealed with a fluorescence quenching sealant, and observed under a fluorescence microscope (DM2000 LED, Leica Camera AG).

### 2.9 Cell counting kit-8 (CCK-8) assay

HCC cells were resuspended in culture medium and seeded in a 96-well plate at a density of 1 × 10^4^ cells per well. The plate was then incubated at 37°C in a 5% CO_2_ atmosphere. Pre-diluted CCK-8 reagent (Dojindo, Shanghai, China) was added, and the plate was further incubated to allow the cells to react. Absorbance at 450 nm was measured for each well using a spectrophotometer (UV-1900, Shimadzu) for 5 consecutive days.

### 2.10 Transwell assay

The upper chamber of the transwell (3422, Corning) was pre-coated with Matrigel (354248, Corning) and then equilibrated at 37°C. After treatment, HCC cells were resuspended in culture medium and seeded in the upper chamber of the transwell at a density of 1 × 10^4^ cells per well in the lower chamber. Culture medium containing 10% serum was added to the lower chamber. The transwell was then incubated in a 37°C, 5% CO_2_ cell culture incubator for 24 h. After incubation, cells that did not migrate through the pores were removed from the upper chamber, while the cells that had migrated were fixed with paraformaldehyde and stained with crystal violet (C0121, Beyotime). Finally, the stained cells were counted under a microscope (DS-Fi3, Nikon).

### 2.11 Statistical analysis

All data were analyzed using GraphPad Prism 9.0 (GraphPad Software, San Diego, CA, USA). Quantitative data were presented as mean ± standard deviation (SD). Comparisons between groups were conducted using Student’s *t*-test or one-way analysis of variance, depending on the number of groups and the distribution of the data. Chi-square tests were used to analyze categorical data. The *p*-value of <0.05 was considered statistically significant.


**Informed consent:** All subjects signed an informed consent.
**Ethical approval:** All procedures were approved by the Ethics Committee of the Affiliated Hospital of the Youjiang Medical University for Nationalities of Ethnic Medicine (ethics review number: YYFY-LL-2013-128).

## 3 Results

### 3.1 The peptide CCAT1-70aa is significantly overexpressed in HCC

**Figure 1:**
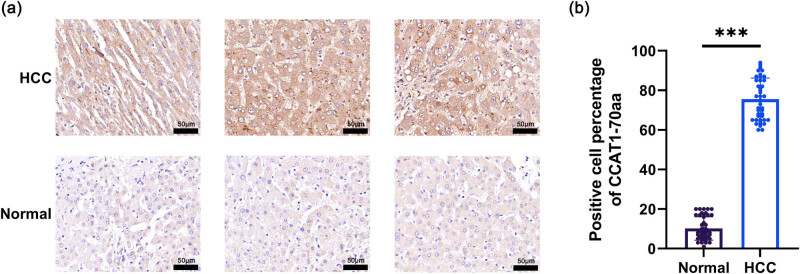
Overexpression of peptide CCAT1-70aa in HCC. (a) Immunohistochemistry of CCAT1-70aa. (b) Quantitative comparison of CCAT1-70aa expression. Means ± SD (*n* = 44, ***P < 0.001).

Immunohistochemistry results demonstrated that CCAT1-70aa is highly expressed in HCC tissues but minimally in the adjacent non-tumorous liver tissues (Figure 1a). Quantitative analysis of immunohistochemistry showed that the positive cell percentage of CCAT1-70aa in HCC tissues is significantly higher than in normal adjacent tissues (*P* < 0.001, Figure 1b). Furthermore, the overexpression of CCAT1-70aa is significantly associated with the tumor pathological stage (*P* = 0.049), serum alpha-fetoprotein (AFP) concentration (*P* = 0.030), and vascular invasion (*P* = 0.020), as shown in Table 1, suggesting that CCAT1-70aa may serve as a potential biomarker for the diagnosis and prognosis of HCC.

**Table 1:** Correlation of CCAT1-70aa expression with characteristics of HCC patients

Characteristics	CCAT1-70aa		*P* value
	Low (*n* = 44)	High (*n* = 44)	
**Age**			0.286
>50	20	25	
≤50	24	19	
**Gender**			0.269
Male	34	38	
Female	10	6	
**Pathologic stage**			0.049*
I + II	37	29	
III + IV	7	15	
**Tumor size (cm)**			0.517
>5	27	24	
≤5	17	20	
**AFP (ng/ml)**			0.030*
≤400	23	13	
>400	21	31	
**Vascular invasion**			0.020*
No	41	33	
Yes	3	11	

AFP: alpha-fetoprotein. *P < 0.05.

### 3.2 Investigation of CCAT1-70aa as a peptide encoded by lncRNA CCAT1

**Figure 2:**
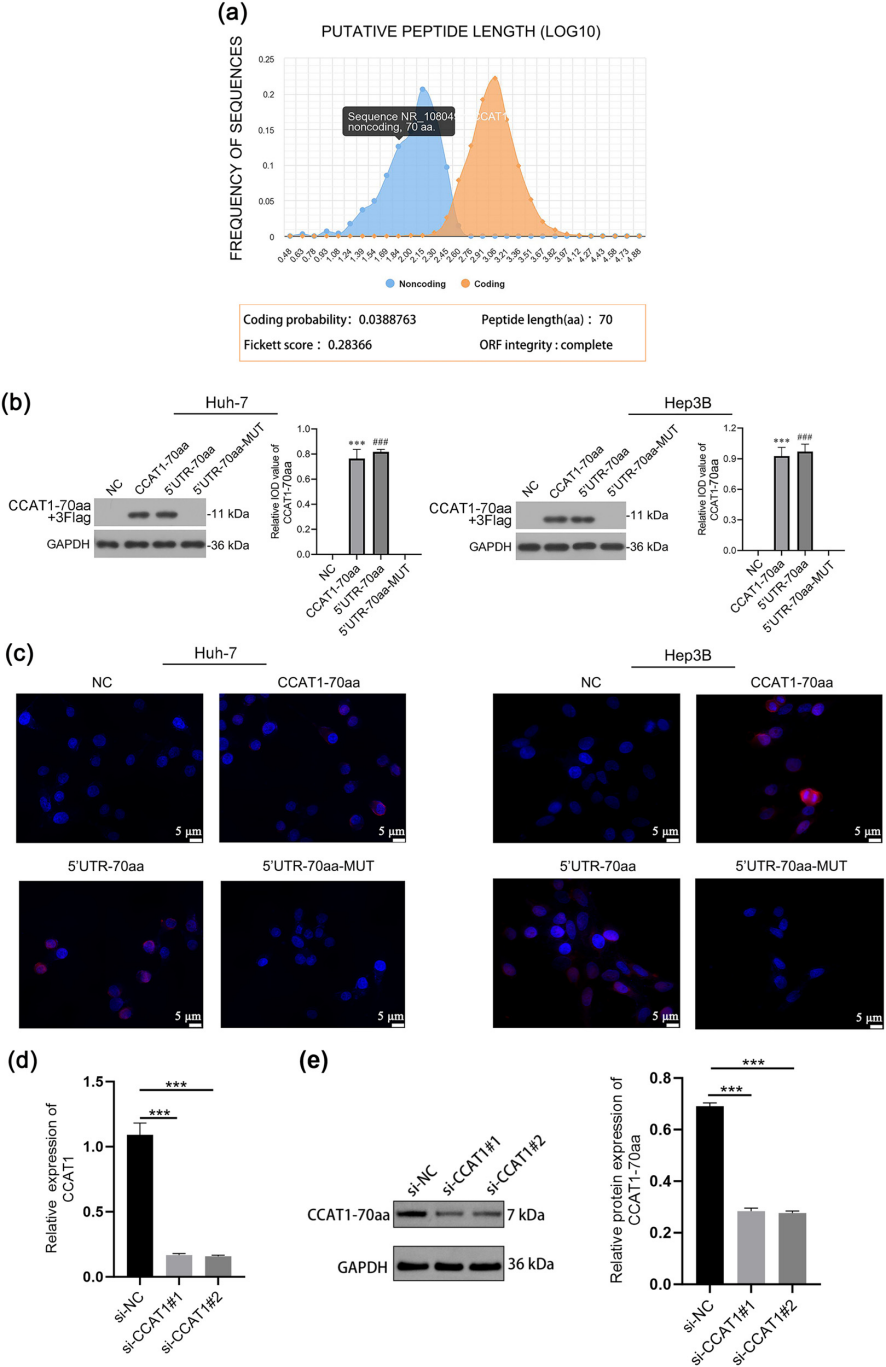
Investigation of CCAT1-70aa as a peptide encoded by lncRNA CCAT1. (a) Open reading frame in lncRNA CCAT1 encoding a peptide was predicted using CPC 2.0 database. (b) Western blot. (c) Immunofluorescence. Blue indicates DAPI staining for nuclear visualization, and red represents the fluorescence signal of CCAT1-70aa, marking the expression location of the CCAT1-70aa protein. (d) RT-PCR analysis of CCAT1 expression after knockdown with si-CCAT1#1 and si-CCAT1#2. (e) Western blot analysis of CCAT1-70aa protein expression after CCAT1 knockdown. Mean ± SD (*n* = 3, ***P < 0.001).

Database annotation suggested that lncRNA CCAT1 possesses an open reading frame capable of encoding a peptide of 70 amino acids (Figure 2a). The expression constructs were tagged with a 3× Flag epitope, and both the Western blot and immunofluorescence analyses were performed using anti-Flag antibodies to specifically detect the CCAT1-70aa peptide. Western blot analysis disclosed no band in the NC group or 5′UTR-70aa-MUT group, whereas a band of 11 kDa was detected in the CCAT1-70aa group and 5′UTR-70aa group (Figure 2b). This corresponds to the expected size of the peptide: 70 amino acids (70 × 110 Da = 7.7 kDa) plus 3× Flag (2.73 kDa) equals approximately 10.4 kDa, which is consistent with an 11 kDa band. Immunofluorescence results further confirmed that the CCAT1-70aa peptide is expressed exclusively in the CCAT1-70aa group and the 5′UTR-70aa group, with expression observed in both the nucleus and cytoplasm (Figure 2c).

To further validate whether CCAT1-70aa is naturally translated from lncRNA CCAT1, CCAT1 knockdown experiments in Huh-7 cells were performed. RT-PCR analysis revealed that silencing CCAT1 using two independent siRNAs (si-CCAT1#1 and si-CCAT1#2) significantly reduced CCAT1 expression compared to the si-NC group (Figure 2d). Western blot analysis further demonstrated that CCAT1-70aa protein expression was significantly decreased following CCAT1 knockdown (Figure 2e). These findings indicate a potential for CCAT1-70aa to be encoded by lncRNA CCAT1.

### 3.3 CCAT1-70aa promotes the proliferation and invasion of Huh-7 and Hep3B cells

**Figure 3:**
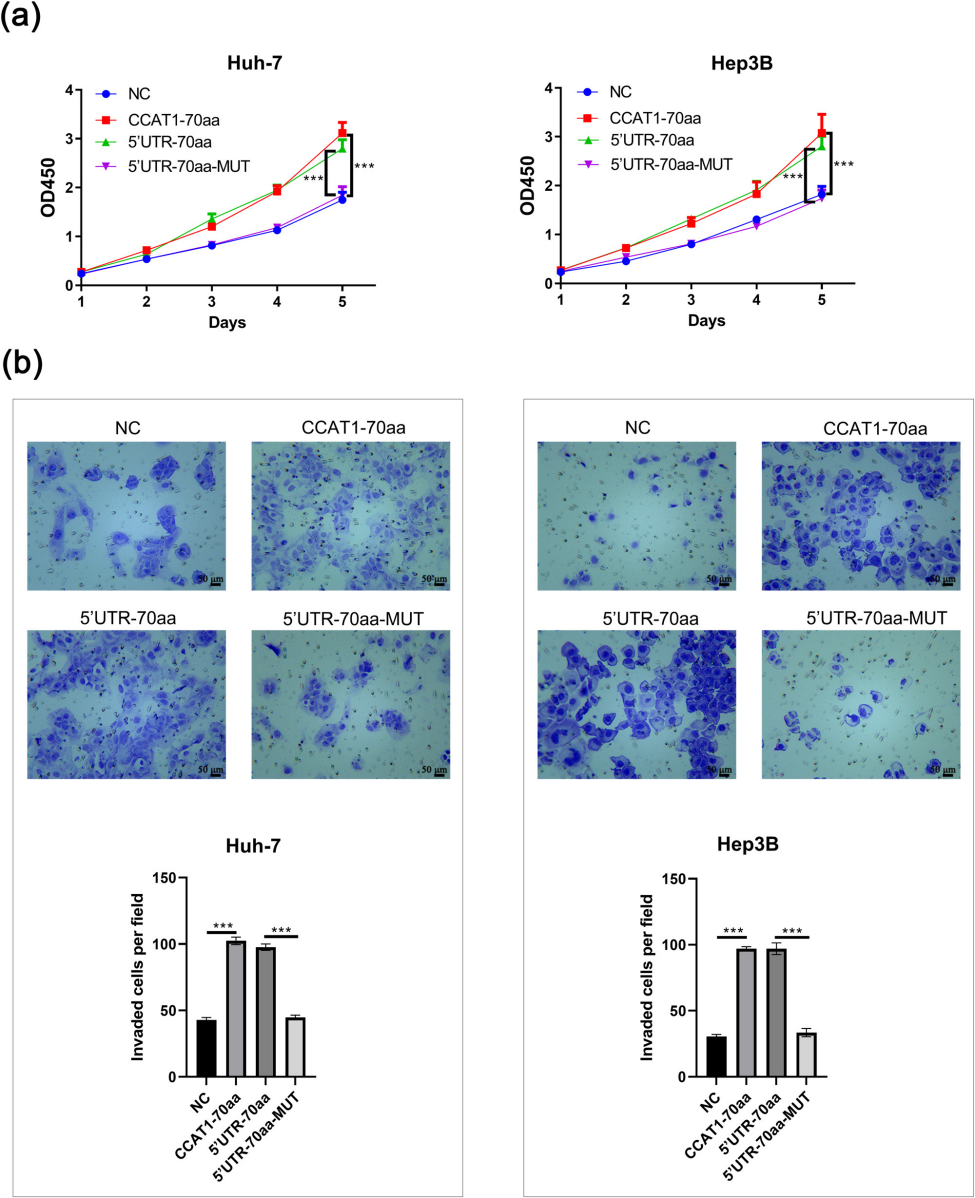
CCAT1-70aa promotes the proliferation and invasion of Huh-7 and Hep3B cells. (a) CCK-8 assay. (b) Transwell assay. Mean ± SD (*n* = 3, ***P < 0.001).

The CCK-8 assay indicated that in Huh-7 and Hep3B cells, the CCAT1-70aa group significantly promoted cell viability compared to the NC group (*P* < 0.001). Similarly, 5′UTR-70aa significantly promoted cell viability compared to the 5′UTR-70aa-MUT group (*P* < 0.001, Figure 3a). Transwell assay results displayed that in Huh-7 and Hep3B cells, compared to the NC group, the CCAT1-70aa group had a significant increase in invasive cells (*P* < 0.001). Compared to the 5′UTR-70aa-MUT group, the 5′UTR-70aa group had a significant increase in invasive cells (*P* < 0.001, Figure 3b). These results suggest that CCAT1-70aa promotes the proliferation and invasion of Hep3B and Huh-7 HCC cells.

### 3.4 CCAT1-70aa promotes Huh-7 cell proliferation and invasion via the MAPK/ERK pathway

**Figure 4:**
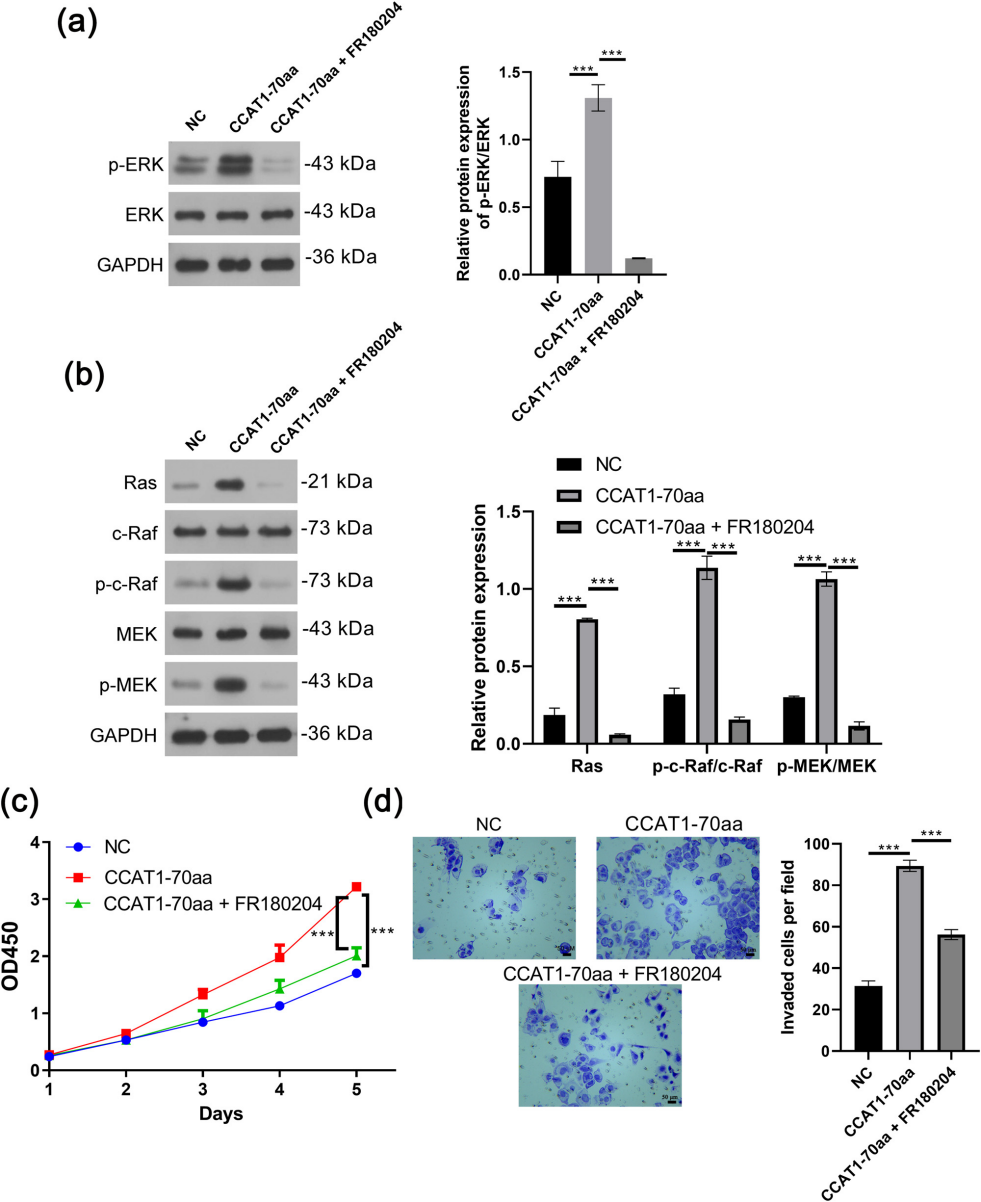
CCAT1-70aa promotes Huh-7 cell proliferation and invasion via the MAPK/ERK pathway. (a) Western blot showing expression of ERK and p-ERK proteins. (b) Western blot showing expression of MAPK/ERK pathway proteins. (c) CCK-8 assay. (d) Transwell assay. Mean ± SD (*n* = 3, ***P < 0.001).

In Huh-7 cells, Western blot analysis revealed that, compared to the NC group, the relative protein expression levels of p-ERK/ERK, Ras, p-c-Raf/c-Raf, and p-MEK/MEK were significantly increased in the CCAT1-70aa group (*P* < 0.001). In contrast, these protein expression levels were significantly decreased in the CCAT1-70aa + FR180204 group compared to the CCAT1-70aa group (*P* < 0.001, Figure 4a and b). CCK-8 assays showed that cell viability was significantly higher in the CCAT1-70aa group than in the NC group (*P* < 0.001), whereas cell viability significantly decreased in the CCAT1-70aa + FR180204 group compared to the CCAT1-70aa group (*P* < 0.001, Figure 4c). Transwell assays indicated a significant increase in the number of invasive cells in the CCAT1-70aa group compared to the NC group (*P* < 0.001), while the number of invasive cells was significantly reduced in the CCAT1-70aa + FR180204 group compared to the CCAT1-70aa group (*P* < 0.001, Figure 4d). These findings suggest that CCAT1-70aa activates the MAPK/ERK pathway and that the ERK inhibitor FR180204 can attenuate the proliferative and invasive effects of CCAT1-70aa in Huh-7 cells, supporting that CCAT1-70aa promotes HCC cell proliferation and invasion via the MAPK/ERK pathway.

## 4 Discussion

HCC is one of the most common and lethal malignant tumors, and the urgent need in clinical practice is to further improve its diagnosis and treatment [27]. Peptides, characterized by their small size, stability, capacity for systemic migration, low immunogenicity, and ability to be secreted into the bloodstream, are promising clinical diagnostic biomarkers [28,29]. Our research suggests that the peptide CCAT1-70aa is significantly overexpressed in HCC, and its high expression is strongly correlated with tumor pathological staging, serum AFP concentration, and vascular invasion. This implies that CCAT1-70aa could potentially serve as an auxiliary diagnostic indicator for HCC to determine the progression of the tumor. It remains to be investigated whether CCAT1-70aa is secreted into the bloodstream and whether its expression in the blood is correlated with the pathophysiology of HCC, which requires further investigation. Moreover, siRNA-mediated silencing of CCAT1 significantly reduced CCAT1-70aa expression. This supports the notion that CCAT1-70aa is translated from lncRNA CCAT1, though further validation is needed to confirm direct translation.

Non-coding RNAs, through the peptides they encode, have emerged as a novel regulatory mechanism playing significant roles in HCC. For instance, a novel peptide encoded by N6-methyladenosine modified circMAP3K4 inhibits apoptosis in HCC cells [30]. The endogenous peptide SMIM30, encoded by LINC00998, promotes the development of HCC by inducing the activation of SRC/YES1 and the MAPK pathway [31]. Additionally, the peptide PINT87aa, encoded by lncRNA, induces cellular senescence in HCC by blocking FOXM1-mediated PHB2 transcription [32]. A novel polypeptide, encoded by the circular RNA ZKSCAN1, has been shown to suppress HCC by degrading mTOR [33]. Similar studies have reported the involvement of peptides encoded by non-coding RNAs in various other tumors [34]. Our study indicates that CCAT1-70aa is potentially encoded by lncRNA CCAT1 and promotes the proliferation and invasion of HCC cells via the MAPK/ERK pathway. This defines a novel functional peptide in HCC, which also provides a reference for studying the function and mechanism of this peptide.

The MAPK family plays crucial roles in a wide range of physiological and pathological processes. ERK, c-Jun N-terminal kinase (JNK), and p38MAPK are typical representatives of MAPKs, which can be activated in HCC [35]. Upon activation by RAS, RAF protein kinases RAF1 and c-Raf are phosphorylated and subsequently activate their dual-specificity protein kinase substrates MEK1 and MEK2 (also known as MAP2K1 and MAP2K2) [36]. Subsequently, MEK1/2 phosphorylates substrates such as ERK, thereby regulating proliferation, differentiation, and migration, among others [37]. Research shows that the MAPK/ERK pathway is extensively involved in the progression regulation of various tumors, and targeted inhibition of the MAPK/ERK pathway has become one of the potential tumor treatment methods [38]. CCAT1-70aa promotes HCC cell proliferation and invasion via the MAPK/ERK pathway. Inhibiting the expression of the CCAT1-70aa peptide and thereby inhibiting the MAPK/ERK pathway may be a potential therapeutic approach for treating HCC. Studies on peptide-drug conjugate-based novel molecular drug delivery systems [39] and novel peptide therapeutic approaches for cancer treatment [40] have opened more possibilities for the application of peptides in clinical cancer treatment.

However, our study has certain limitations. First, we could not conduct *in vivo* animal studies to verify the function and mechanism of CCAT1-70aa. Second, we lack further molecular biological experiments to confirm how the CCAT1-70aa peptide regulates the MAPK/ERK pathway through interaction. Additionally, although our study explored the relationship between the CCAT1-70aa peptide and the clinical pathology of HCC, survival analysis was not conducted, and the clinical samples used for verification were limited to formalin-fixed paraffin-embedded tissues, which restricts our ability to perform more analyses on HCC tumors versus normal adjacent tissues. As a result, we were unable to confirm the expression of CCAT1-70aa in the context of surrounding normal tissues. Furthermore, we did not include a group treated with only FR180204, as the focus of our study was on the effects of CCAT1-70aa. However, this absence does represent a limitation in evaluating the specific effects of FR180204 alone. Therefore, further validation is needed with more clinical samples from multiple institutions.

In conclusion, our study reveals that CCAT1-70aa, a peptide composed of 70 amino acids potentially encoded by lncRNA CCAT1, is significantly overexpressed in HCC. High expression of CCAT1-70aa correlates significantly with tumor pathological staging, serum AFP concentration, and vascular invasion. CCAT1-70aa promotes the proliferation and invasion of HCC cells via the MAPK/ERK pathway. This provides fresh insights and avenues for the diagnosis and treatment of HCC.
